# A multilevel intervention to promote HPV vaccination among young adults in Texas: protocol for a randomized controlled trial

**DOI:** 10.1186/s12889-024-18828-9

**Published:** 2024-06-05

**Authors:** Qian Lu, Lenna Dawkins-Moultin, Dalnim Cho, Naomi Q. P. Tan, Suellen Hopfer, Yisheng Li, Lois Ramondetta, Yusi Xu, Di Lun, Minxing Chen

**Affiliations:** 1 Department of Health Disparities Research, The University of Texas MD Anderson Cancer Center, 1400 Pressler St. Unit 1440, Houston, TX 77030-3906 USA; 2 Department of Health Services Research, The University of Texas MD Anderson Cancer Center, Houston, TX USA; 3 Rutgers Cancer Institute of New Jersey, and Division of Medical Oncology, Robert Wood Johnson Medical School, Rutgers University, New Brunswick, NJ USA; 4 University of California Irvine, Irvine, CA USA; 5 Department of Biostatistics, The University of Texas MD Anderson Cancer Center, Houston, TX USA; 6 Department of Gynecologic Oncology and Reproductive Medicine, Division of Surgery, The University of Texas MD Anderson Cancer Center, Houston, TX USA

**Keywords:** Papillomavirus vaccines, Young adults, Narrative persuasion, Randomized controlled trial, Multilevel intervention, Psychosocial intervention, Video narratives, Written narratives, Access, Version 1.

## Abstract

**Background:**

Human papillomavirus (HPV) infections can cause cancers of the cervix, vagina, vulva, penis, anus, and oropharynx. The most recently approved HPV vaccine, Gardasil-9, protects against HPV infection and can prevent HPV-associated invasive cancers. However, Gardasil-9 is one of the most underused vaccines in the US today. Young adults are at risk for HPV infection, but many are not vaccinated. This study uses a randomized controlled trial (RCT) to test an innovative multilevel intervention to increase HPV vaccination rates among young adults. In this paper, we describe the research protocol.

**Methods:**

The study uses a two by three factorial design. A total of 1200 young adults in Texas, age 18–26 years, who have not been previously fully vaccinated against HPV will be randomly assigned to one of six conditions to receive: (1) standard CDC information about HPV vaccination (control); (2) video narratives about HPV vaccination; (3) written narratives about HPV vaccination; or (4–6) enhanced access to HPV vaccine combined with (4) standard CDC information, (5) video narratives, or (6) written narratives. The two primary outcomes are the rate of HPV vaccination initiation by 3-month follow-up and rate of HPV vaccination completion by 9-month follow-ups. We will determine the impact of the individual level intervention (i.e., persuasive narratives through video or written format), the systemic level intervention (i.e., enhanced access to HPV vaccines), and the combination of both levels, on HPV vaccination initiation and completion. We will also use purposive sampling to select participants to take part in semi-structured interviews/focus groups to better understand the mechanisms of the intervention.

**Discussion:**

Recruitment and data collection began in March 2022. We expect to complete data collection by March 2026. We expect that narratives, enhanced access, and the combination of both will improve HPV vaccination initiation and completion rates among young adults. If proven successful, these individual- and system-level interventions can be easily disseminated in regions with low HPV vaccination rates to improve HPV vaccination, and ultimately decrease HPV-related cancer burden.

**Trial Registration:**

NCT05057312.

**Supplementary Information:**

The online version contains supplementary material available at 10.1186/s12889-024-18828-9.

## Introduction

Nearly all sexually active individuals will be infected with the human papillomavirus (HPV) at some point in their lives, making HPV the most common sexually transmitted infection in the US [1]. Persistent HPV infection with high-risk HPV types can lead to the development of cancers of the cervix, vagina, and vulva in women, cancers of the penis in men, and anogenital warts and cancers of the anus and oropharynx in both women and men [2]. In the US, the lifetime medical cost of treating HPV-related diseases from infections acquired in just one year is estimated to be $774 million [3]. Gardasil-9, the most recently approved HPV vaccine, protects against nine types of HPV and can prevent up to 90% of HPV-related cancers from developing if given before exposure to the virus [4]. In their 2012–2013 annual report, the President’s Cancer Panel highlighted HPV vaccine uptake as a public health priority for the primary prevention of HPV-related cancers [4].

The Centers for Disease Control and Prevention (CDC) recommend that the HPV vaccine be given routinely at 11–12 years of age [2]. Starting in 2019, for individuals not vaccinated at age 11–12 years, the CDC recommends catch-up vaccination through age 26 years [2]. The recommended schedule for catch-up vaccination is three doses, with the second dose given 1 to 2 months after the first dose, and the third dose given 6 months after the first dose [2]. Despite the recommendation, HPV vaccination rates among young adults aged 18–26 years have been lagging behind. Data from the National Health Interview Survey (2018) show that among all young adults, 53.6% of women and 27% of men reported receiving at least one dose of the HPV vaccine, and 35.3% of women and 9% of men completed the vaccine series [5]. Among young adults 18 − 26 years, 39.9% received one or more doses of the HPV vaccine and 21.5% completed the vaccine series [5]. An analysis of the 2015–2017 Behavioral Risk Factor Surveillance System (BRFSS) data also found that the Southern region of the US, where Texas is located, had the lowest proportion of young adults initiating and completing the HPV vaccine series among all regions [6, 7].

Young adults, and in particular college students, are a priority target population for catch-up HPV vaccination efforts because of their increased risk for HPV infection. The National College Health Assessment II 2018 data found that only 58.7% of students had undergone HPV vaccination [8]. Among approximately 20 million students attending college [9], it is estimated that more than 7 million need catch-up HPV vaccination. Hence, there is an urgent need to improve HPV vaccination rates on college campuses for the primary prevention of HPV-related cancers. The literature shows college students face numerous barriers to HPV vaccination including lack of information or knowledge [10], low perception of susceptibility to HPV infection [10, 11], concerns about vaccine safety [10], concerns about cost [7], concerns about parental disapproval [12], difficulty accessing HPV vaccination providers [13], and time constraints [14]. On the other hand, facilitators of HPV vaccination among college students include the desire to prevent cancer [15], provider recommendation [15], and availability of the vaccine on campus [16]. Because these barriers and facilitators are at both the individual and system level [10, 11, 17, 18], it is imperative that interventions to increase HPV vaccination rates among college students are multilevel and address systemic barriers to HPV vaccination.

However, a systematic literature review of publications up to December 2017 identified only eight randomized controlled trials (RCTs) to improve HPV vaccination rates among college students [19]. Seven of the RCTs did not find differences in HPV vaccine uptake between intervention and control groups [19], while only one found benefits of the intervention in increasing self-reported uptake of one dose of the HPV vaccine [20]. Furthermore, six RCTs measured vaccine uptake of at least one dose and only one RCT measured vaccine uptake and completion [19], indicating a need for longitudinal follow-up to assess the impact of the interventions on HPV vaccination completion. Moreover, most of the interventions had samples that were predominantly non-Hispanic white (NHW) females [19]. Only one RCT included males, with majority of existing interventions focusing on female college students [21]. As the HPV vaccine for males was approved later than for females, young adult men already experience disproportionately lower rates of vaccination initiation and completion than young adult women [22]. Our recent review also showed that college men reported lower knowledge of HPV and HPV vaccination than college women and that racial/ethnic minority college women were less likely to be vaccinated than were NHW college women [23]. These results suggest the importance of including young adult men and members of racial/ethnic minorities in HPV vaccination interventions to avoid exacerbating existing health disparities.

We planned an RCT to test the impact of a multilevel intervention for college students in Texas, where HPV vaccination rates are low [24, 25], and to identify how and for whom the intervention is effective. The intervention will test two major components: (1) narrative persuasion (individual level) and (2) enhanced access to the HPV vaccine (systemic level). A prior study found that female college students in the US who viewed video narratives from the perspective of peers and experts were twice as likely to obtain HPV vaccination compared to the control groups [20]. Building on this work, we conducted a pilot study using tailored written narratives from the perspective of peers and experts. The results suggested that the tailored written narratives have the potential to increase HPV vaccination intentions among young adults as well [26]. Qualitative data from our pilot study also suggested the need to overcome HPV vaccination access barriers at a system level [26]. These barriers include lack of access to the HPV vaccine on college campuses, difficulty navigating insurance, lack of insurance, lack of time to figure out where to get vaccinated, and lack of transportation [26]. These findings suggest that individual-level narrative interventions are potentially effective but that a system-level intervention to enhance access to HPV vaccines is needed to address barriers at the system-level [26]. Accordingly, the overall aim of this study is to assess the impact on HPV vaccination initiation and completion of a multilevel intervention that combines narrative persuasion and enhanced access to HPV vaccination. To our knowledge, this study is the first large-scale multilevel intervention to test the combination of narrative persuasion and enhanced access to address individual and system-level barriers to receiving HPV vaccination among college students.

## Methods

This study protocol conforms with the Standard Protocol Items: Recommendations for Interventional Trials (SPIRIT) guidelines (see Table 1 for the SPIRIT flow diagram showing the schedule of enrolment, interventions, and assessments and Additional File 1 for the completed SPIRIT checklist) [27].

### Aims

The primary aim of this RCT is to evaluate the impact of addressing individual and system levels of influence on HPV vaccination initiation and completion rates **(Aim 1)**. First, we hypothesize that narratives (video or written) combined with access to the HPV vaccine will both improve HPV vaccination rates over standard CDC HPV information alone (control) (*Hypothesis 1.1).* Second, we hypothesize that narratives (video or written) will improve HPV vaccination rates compared to receiving the control, which is the CDC HPV information *(Hypothesis 1.2).* Third, we hypothesize that enhanced access to the HPV vaccines will increase HPV vaccination rates over vaccination rates achieved with usual care (no enhanced access) *(Hypothesis 1.3)*. The secondary aim of this RCT is to understand the underlying mechanisms of the intervention and explore characteristics of individuals who benefit from the intervention **(Aim 2)**. Guided by the behavioral theories (Theory of Planned Behavior [28], Social Cognitive Theory [29, 30], and Health Belief Model [31]), We hypothesize that theorized factors (i.e., HPV vaccination knowledge, attitudes, perceived social norms, safety concerns, and perceived susceptibility) will mediate group differences in HPV vaccination outcomes (Hypothesis 2.1). In addition, we have two research questions: *RQ1.* For whom is the intervention most effective and why? *RQ2.* What critical factors facilitate or hinder implementation of the intervention based on stakeholders’ perspectives?

### Design

The study utilizes a 2-by-3 factorial design (enhanced access to HPV vaccine: yes vs. no; narratives: video vs. written vs. none) (Table 1). The no-narrative groups will receive standard CDC information presented in a didactic format. Participants will be randomly assigned to one of six groups: (1) enhanced access and standard CDC information (control), (2) enhanced access and video narratives, (3) enhanced access and written narratives, (4) standard CDC information only (control), (5) video narratives only, and (6) written narratives only. The two primary outcomes will be rate of HPV vaccination initiation by 3-month follow-up and rate of HPV vaccination completion (receiving 3 doses of the HPV vaccine) by 9-month follow-up. After the 9-month follow-up, we will conduct semi-structured interviews or focus groups to understand what made the intervention work in individuals who received at least one dose of the vaccine and what made the intervention not work in individuals who did not initiate.

**Table 1:** Factorial design

	No Narratives (CDC Information)	Video Narratives	Written Narratives
Enhanced Access	Group 1	Group 2	Group 3
No Enhanced Access	Group 4	Group 5	Group 6

### Participants and eligibility criteria

The inclusion criteria are: (1) age 18–26 years; (2) ability to read and understand English; (3) self-reported as not yet having received any HPV vaccine injections; (4) access to a smart phone, tablet or computer that is connected to the internet; and (5) currently enrolled in one of the participating schools with an anticipated continuous enrollment of at least 9 months. The exclusion criteria are: (1) being pregnant or (2) having a life-threatening allergy to any component of the HPV vaccine.

### Settings and recruitment

We will recruit 1200 women and men in total. Participants will be recruited primarily from universities in the Houston metropolitan area— University of Houston, which has a large Hispanic student population; Texas Southern University, a historically Black university; Sam Houston State University, a Hispanic-serving institution; and Rice University, which has a large White and Asian American student population. Recruitment strategies will focus on mass e-mailing, posting fliers, in-person campus recruitment using student volunteers and staff recruiters, engaging student organizations and research labs, and in-class study announcements. We will also use various promotional items (e.g. bottled water, school supplies, sunscreen, etc.) that carry the study information to advertise. In addition, we will discuss recruitment strategies with student advisory boards and be guided by the input from these students. Participants will receive gift cards for completing surveys and participating in qualitative interviews. Participation retention is promoted through incentives after each study task, automated email reminders, and reminders from study staff via texts or phone calls.

### Description of intervention

#### Narrative conditions

Prior work suggests that narratives that include multiple sources (e.g. peers and experts) are more persuasive than a single source [20]. Participants in the video narrative groups will watch four videos – two from the perspectives of peers and one each from the perspective of a doctor and a cancer survivor. Participants in the written narrative groups will read three narratives – one each from a peer, doctor, and cancer survivor. The videos and written narratives are matched to have similar content and word count with each narrative taking about 1.5 min to read or view. Each narrative is tailored for males or females by changing the narrators (e.g. male narratives have male narrators) and other relevant characteristics (e.g. cancer type – oropharyngeal cancer for men, cervical cancer for women). The written narratives from peers are further tailored for sexual activity status (i.e. sexually active versus inactive). The narrative intervention contents will be automated and hosted on Research Electronic Data Capture (REDCap), a data collection platform. Participants will be shown the narratives for their tailored group based on their self-reported biological sex at birth (male vs. female) (video narrative groups) and sex and sexual activity status (written narrative groups). We will include adherence checks after the video and written narratives. Participants will be asked to summarize what they have learned after the interventions to test if they viewed the materials.

#### Non-narrative control conditions

Participants in the non-narrative conditions will receive standard CDC information about HPV and the HPV vaccine [32]. Participants will be asked to summarize what they have learned after viewing the CDC information to test if they viewed the materials.

#### Enhanced Access to HPV Vaccine conditions

Enhanced access will be in the form of patient navigation, on-campus vaccine clinics, and financial assistance. At the end of the narrative intervention/CDC information sessions, participants in enhanced access condition will receive information about vaccination options automated through REDCap based on their self-reported insurance plan. Students with private insurance or Medicaid will be informed of dates for on-campus HPV vaccine clinics organized by the study staff in partnership with a local pharmacy and be offered the opportunity to pre-register to attend. Students who are uninsured will be assisted to apply for the Merck Vaccine Patient Assistance Program and will be navigated to the school health center to receive HPV vaccination. Participants will receive email reminders before the initial clinics. After participants complete the initial dose of the HPV vaccine, they will receive reminders to register for the second and third doses. Participants who are unable to attend the on-campus clinics for any reason or whose second or third dose are due during a school holiday will be contacted by study staff and given the option to get vaccinated at a participating pharmacy near them or be rescheduled for the next on-campus clinic. Participants will not have any out-of-pocket costs for receiving HPV vaccination.

#### No enhanced access (Usual Care) condition

Participants in the conditions without enhanced access to HPV vaccine will be sent a list of community HPV vaccine providers via email. At the end of the 9-month follow-up, participants in the usual care condition who reported that they did not initiate or complete vaccination will be invited to attend the upcoming on-campus clinics to start or complete the series.

### Sample size calculation

Our sample size calculations are mainly based on hypothesis testing for both vaccination initiation and vaccination completion in Aim 1, with a two-sided alpha of 0.005 (= 0.05/10) (with a Bonferroni adjustment) for each test. This adjustment is used because Aim 1 has 10 tests in total, including five comparisons specified in hypotheses for each of the two HPV vaccination outcomes. The five comparisons are for two simple effects (i.e., written/video narrative + access vs. control), two marginal effects (i.e., written/video narrative vs. no narrative), and a main effect (access vs. no access). The assumed HPV vaccination rates for these comparison groups are calculated based on the expected vaccination rates inferred from the literature [20, 21, 33, 34] for the six groups (i.e., control, written narratives, video narratives, access only, access + written, and access + video): 12%, 20%, 22%, 20%, 40%, and 42% at 3-month follow-up for initiation, and 4%, 10%, 11%, 15%, 30%, and 32% at 9-month follow-up for completion, respectively. Post-attrition sample sizes used for power analysis represent the worst-case scenario and thus provide conservative estimates. Assuming 15% and 20% attrition at 3- and 9-month follow-ups, a total sample size of 1200 at baseline yields 1020 for analysis of vaccination initiation (1200*85%=1020) and 960 for completion (1200*80%=960), respectively. Sample sizes available per group for each comparison/test (written/video narratives + access vs. control, written/video narrative vs. no narratives, access vs. no access) are 170, 340, and 510 for HPV vaccination initiation and 160, 320, and 480 for HPV vaccination completion, respectively. Power analysis using nQuery 7.0 [35] shows that these sample sizes will have excellent power (82%~99%) to detect group differences in each of the 10 tests.

### Randomization

The randomization, which will be conducted using REDCap, will be stratified by sex, sexual activity status, and race/ethnicity (Black, Asian, White, Hispanic, and Other) so that the joint distribution of sex, sexual activity status, and race-ethnicity is equal across conditions. Participants and researchers will be blinded to the condition assignment except for those who deliver the intervention (providers and personnel coordinating the vaccination). Participants will be told the study is testing HPV messages but they will not be told what the different conditions are, nor their condition assignment. Specifically, we will set up stratified randomization (based on sexual activity status) into the six intervention groups in Table 2, within each of the 10 sex by racial or ethnic subgroups of participants.

### Measures

Guided by behavioral theories (health belief model [31], social cognitive theory [29, 30], theory of planned behavior [28]), we carefully selected measures from extant HPV vaccination studies among college students and young adults. Table 2 shows the measures that are included in this study and their measurement time-points.

#### HPV vaccination initiation and completion (primary outcomes)

HPV vaccination initiation will be determined by self-report at follow-ups, and verified by medical record review. HPV vaccination completion by the 9-month follow-up will be determined by self-report, and verified by medical record review. Based on CDC’s HPV vaccine guidelines [2], participants can complete the HPV vaccine series in 6 months. Therefore, we have added a 6-month follow-up is to capture completion data for participants who might complete the vaccine series quickly and to minimize the loss of completion data as a result of attrition at 9-month follow-up. That is, vaccine completion by the 9-month follow-up will be defined by the self-reported completion status at the 9-month follow-up or the 6-month follow-up if the report is missing at 9-month follow-up due to attrition. At all follow-ups, students who report vaccination will be given the option to sign a medical record release or take a picture of their HPV vaccine record and upload to REDCap.

#### Secondary outcomes

Secondary outcomes are self-efficacy to be vaccinated for HPV and intention to be vaccinated after the intervention. The intention measure has two items that are typically used in HPV vaccine research and are based on the theory of planned behavior [28]. The questions query participants’ intention to initiate vaccination within 3 months and to complete the HPV vaccine series. The self-efficacy measure (3 items) [20, 36] was adapted from previous HPV studies among college students and young adults.

#### Mediators

We include 5 mediating variables: (1) *Knowledge* (8 items, one of which was tailored based on sex) will be assessed with true/false responses, and the percentage of correct responses will be calculated; (2) *HPV Vaccine Attitude* (3 items) measures attitudes towards the HPV vaccine; (3) *Perceived Social Norms* (Injunctive Norms − 3 items; Descriptive Norms − 2 items, tailored based on sex) measures participants’ perceptions of whether others in their social network would approve of the HPV vaccine and are based on scales with high item reliability among college students [37]; (4) *Safety Concerns* related to the HPV vaccine (5 items) [10, 38, 39]; and (5) *Perceived Susceptibility and Severity of HPV Infection* (6 items) [10] are adapted from previous HPV studies among college students and young adults.

#### Covariates

Potential covariates are demographic variables (age, sex, year in college, ethnicity, insurance coverage, household income, parents’ level of education) and sexual history (sexually active or not).

#### Data quality

To ensure data quality, we will include two attention check questions: one in the first half of the questionnaire and the other in the last half. An example of the attention check questions is “On this item, please answer “Strongly Disagree.” The questions will help verify if participants are reading the questions. Participants who answer one or both attention check questions incorrectly will be removed from the data analysis.

### Data collection

Participants who are eligible and agree to participate in the study will be asked to complete an informed consent form on REDCap. After signing the informed consent form, participants will complete online questionnaires on REDCap. Participants will first complete a brief demographic survey (i.e. sex, sexual activity status) in order to receive tailored narratives. Participants will then complete a baseline survey (T0), view the materials for their assigned group (described above), and complete the post-intervention survey (T1). At 3-, 6-, and 9-month, follow-up surveys will be administered. These will be sent as links to participants via email or text message (depending on the preference of the participants). At the 3-, 6- and 9-month follow-ups, all participants who report initiating or completing the HPV vaccine series will be asked to consent to releasing their medical records on HPV vaccination from the clinic, pharmacy, or doctor’s office where they were vaccinated.

#### Qualitative interviews

After the 9-month follow-up, we will purposively select participants to take part in focus groups or in-depth interviews to understand (1) what made the intervention work in individuals who received at least one dose of the vaccine and (2) what made the intervention not work in individuals who did not initiate HPV vaccination. Focus groups and/or interviews will last approximately 60–90 min and will continue until data saturation is reached. Focus groups/interviews will be conducted virtually or in-person depending on participants’ schedule and preference.

### Data analysis

Prior to the inferential procedures, extensive descriptive statistical analyses will be performed on all variables of interest. Distributional assumptions will be evaluated, and if indicated, normalizing (such as log) transformations or robust procedures will be employed (e.g., for the continuous mediating variables). Appropriate procedures to assess the validity and reliability of the questionnaires will be used. As a general strategy for our primary analysis, an intention-to-treat (ITT) approach will be used.

#### Aim 1

Logistic regression analysis will be used to test the five comparisons on both the HPV vaccination initiation and completion rates (binary: yes vs. no), controlling for the stratification factors (gender, sexual activity status, and race/ethnicity) and additional covariates as appropriate [40]. While we do not anticipate a strong cluster (university) effect, we will explore the above comparisons using generalized linear mixed models (GLMMs) with a binomial outcome distribution with a logit-link function to account for potential within-university correlations. Each of the hypotheses in Aim 1 will be tested at a 2-sided 0.005 significance level to ensure statistical reproducibility by controlling the overall type I error rate at 0.05.

#### Aim 2

For *hypothesis 2.1*, we will examine if the impact of the intervention on outcomes at the 3-, 6-, and 9-month follow-ups are achieved through improved hypothesized mediators at the post-intervention and 3-month follow-up, respectively. We will use linear regression analysis in the intervention-mediator (a) path, and a logistic regression analysis in the intervention + mediator-outcome (b) path; the indirect (or mediation) effect will be defined by the product of the appropriate coefficients from the a and b paths [41, 42]. 95% bootstrap confidence intervals will be calculated to assess the significance of the indirect effects, using existing macros [43]. Both single- and multiple-mediator models will be tested [42]. To address *RQ1*, we will conduct analyses similar to those described in Aim 1 but focus on testing the interaction effects between the intervention condition and gender, a potential moderator of the intervention effect. Similar to the Aim 1 analyses, we will explore using linear mixed models (LMMs) for the path a analyses and GLMMs for the path b analyses to account for potential cluster (university) effects. All analyses performed in Aim 2 will use a 2-sided 0.05 significance level for hypothesis-generating (rather than confirmatory finding) purposes.

#### Handling of missing data and attrition

Participants in the study may be lost to follow-up, resulting in missing data. Consistent with the ITT approach to our primary analysis, we designate a participant’s group based on his/her randomization assignment despite potential group switch. We will impute missing data using an appropriate multiple imputation approach, assuming a missing-at-random mechanism [44]. Additional analyses will be conducted based on the observed data, which will give asymptotically unbiased estimates of the effects of interest, provided that missingness depends only on the observed variables included in the model. However, missing data may not be missing at random (e.g., participants who do not initiate or complete vaccination may choose to leave the study early); thus, we will conduct sensitivity analyses assuming different missing data mechanisms, especially when attrition is moderate or high (e.g., > 20%) and unbalanced across intervention conditions. We will explore pattern-mixture or selection models to account for potential missing-not-at-random (MNAR) mechanisms [44]. Similar results from the sensitivity analyses would strengthen our study findings.

To answer RQ1 and RQ2, we will conduct a content analysis of the qualitative data (interviews, meeting minutes, work logs, etc.) using a phenomenological method and a systematic approach to capture the themes. Interview data from students will be content analyzed in the context of gender, sexual activity status, and ethnic differences to inform future strategies to refine HPV vaccination intervention. Using inductive methods will shed light on successes and lessons learned in this study and help to clarify factors that facilitate or hinder implementation and dissemination of the intervention.

### Ethics and dissemination

Ethics approval was obtained from the University of Texas MD Anderson Cancer Center (Houston, Texas) Human Subjects Protection Committee, the institutional review board (IRB) (Protocol 2020 − 1142). Written informed consent will be required before participation. Participants are free to withdraw from the study at any time without any penalty. All important protocol modifications will be submitted to the IRB for approval prior to being implemented. The study was deemed exempt from data safety and monitoring by an independent board as it is low risk. The Principal Investigator and IRB are responsible for monitoring and reporting of any adverse events during the study. All participants’ identification records will be kept confidential in a secure file with password protection. Each participant will be assigned an identification number that will be used on all documents and data files. No data will be associated with personal identifiers. Only the IRB-approved research team members will have access to identifiable participant data during the study. The outcomes of the study will be disseminated to the scientific community through presentations at academic conferences and publications in peer-reviewed journals.

**Table 2:** Schedule of enrolment, interventions, and assessments for the NO-HPV-4-ME Intervention (SPIRIT Flow Diagram)

	Study Period							
Timepoint	Enrollment	Randomization	T0^a^	Intervention	T1^a^	T2^a^	T3^a^	T4^a^
**Enrolment:**								
Eligibility screen	X							
Informed consent	X							
Randomization		X						
**Interventions**:								
CDC Information + No Enhanced Access				X				
CDC Information + Enhanced Access				X				
Video Narratives + No Enhanced Access				X				
Video Narratives + Enhanced Access				X				
Written Narratives + No Enhanced Access				X				
Written Narratives + Enhanced Access				X				
**Asessments**:								
**Primary Outcomes**:								
HPV Vaccine Initiation						X	X	X
HPV Vaccine Completion							X	X
**Secondary Outcomes**:								
Self-Efficacy			X		X	X	X	
Intention to Initiate Vaccination			X		X	X^b^	X^b^	X^b^
Intention to Complete 3 Doses of the HPV Vaccine			X		X	X	X	X^c^
**Mediators**:								
Knowledge			X		X	X		
Attitudes			X		X	X		
Perceived Social Norms			X		X	X		
Safety Concerns			X		X	X^b^		
Perceived Susceptibility and Severity			X		X	X	X	

^a^T0 = Baseline survey; T1 = Post-intervention survey; T2 = 3-month follow-up; T3 = 6-month follow-up; T4 = 9-month follow-up

^b^These questions were only administered to participants who had not yet initiated the vaccine series

^c^These questions were only administered to participants who had not yet completed the vaccine series

## Discussion

Few evidence-based multilevel behavioral interventions are available to promote HPV vaccination for young adults. To the best of our knowledge, this project is the first RCT to test a multi-level intervention in young adults from multiple ethnic groups. In this RCT, we will determine the impact of individual-level intervention (narrative persuasion) and system-level intervention (enhanced access to HPV vaccination) on HPV vaccine initiation and completion at 3-, 6-, and 9-month follow-ups. This program has the potential to be widely disseminated on college campuses. Recruitment began in March 2022, and we expect to complete all data collection by March 2026. We expect to complete data management by August 2026 and submit the main study results for publication by 2027.

The design of this study has both strengths and limitations. The intervention is innovative, as it is a multilevel intervention addressing both individual- and system-level barriers to HPV vaccination. The pharmacy partnership is also an accessible model for future dissemination. Other strengths of the study are the use of a factorial design, which will delineate the factors/levels that work best, and the large sample size drawn from multiple universities and composed of multiethnic groups of students. Finally, the investigation of mediators of the intervention will further help us understand the underlying mechanisms for the impact of the intervention and allow us to further tailor the intervention.

The study has a few limitations. The intervention will be tested in 4-year colleges, so the results may not be generalizable to other young adults who do not attend college or who attend a 2-year or community college. In addition, the intervention will be conducted in English, and therefore may not be directly applicable to those with limited English proficiency. Finally, the video narratives were tailored based on sex at birth and featured heterosexual characters. Hence, the effects on sexual and gender minorities may be limited. Even with these limitations, the intervention has promise of high impact for cancer prevention. If the program is demonstrated to improve HPV vaccination rates among young adults, it may be disseminated across Texas and the U.S., which will ultimately help reduce the burden of HPV-related cancers among young adults.

### Electronic supplementary material

Below is the link to the electronic supplementary material.

Supplementary Material 1

## Data Availability

No datasets were generated or analysed during the current study.

## Abbreviations

HPV: Human papillomavirus
RCT: Randomized controlled trial
